# Dynamic species classification of microorganisms across time, abiotic and biotic environments—A sliding window approach

**DOI:** 10.1371/journal.pone.0176682

**Published:** 2017-05-04

**Authors:** Frank Pennekamp, Jason I. Griffiths, Emanuel A. Fronhofer, Aurélie Garnier, Mathew Seymour, Florian Altermatt, Owen L. Petchey

**Affiliations:** 1 Department of Evolutionary Biology and Environmental Studies, University of Zurich, Winterthurerstrasse 190, CH-8057 Zurich, Switzerland; 2 Department of Animal and Plant Sciences, University of Sheffield, Western Bank, Sheffield, S10 2TN, United Kingdom; 3 Department of Aquatic Ecology, Eawag: Swiss Federal Institute of Aquatic Science and Technology, Überlandstrasse 133, CH-8600 Dübendorf, Switzerland; 4 Molecular Ecology and Fisheries Genetics Laboratory, School of Biological Sciences, Bangor University, Deiniol Road, Gwynedd LL57 2UW, United Kingdom; Tianjin University, CHINA

## Abstract

The development of video-based monitoring methods allows for rapid, dynamic and accurate monitoring of individuals or communities, compared to slower traditional methods, with far reaching ecological and evolutionary applications. Large amounts of data are generated using video-based methods, which can be effectively processed using machine learning (ML) algorithms into meaningful ecological information. ML uses user defined classes (e.g. species), derived from a subset (i.e. training data) of video-observed quantitative features (e.g. phenotypic variation), to infer classes in subsequent observations. However, phenotypic variation often changes due to environmental conditions, which may lead to poor classification, if environmentally induced variation in phenotypes is not accounted for. Here we describe a framework for classifying species under changing environmental conditions based on the random forest classification. A sliding window approach was developed that restricts temporal and environmentally conditions to improve the classification. We tested our approach by applying the classification framework to experimental data. The experiment used a set of six ciliate species to monitor changes in community structure and behavior over hundreds of generations, in dozens of species combinations and across a temperature gradient. Differences in biotic and abiotic conditions caused simplistic classification approaches to be unsuccessful. In contrast, the sliding window approach allowed classification to be highly successful, as phenotypic differences driven by environmental change, could be captured by the classifier. Importantly, classification using the random forest algorithm showed comparable success when validated against traditional, slower, manual identification. Our framework allows for reliable classification in dynamic environments, and may help to improve strategies for long-term monitoring of species in changing environments. Our classification pipeline can be applied in fields assessing species community dynamics, such as eco-toxicology, ecology and evolutionary ecology.

## Introduction

Society is presently in the midst of an automation revolution that was initiated in the middle of the 20th century by the invention of the Turing machine. Tasks once performed by humans are steadily being relinquished to computers that are more efficient at tedious jobs than their human counterparts. Likewise, ecologists are increasingly relying on semi- or fully automated monitoring systems to collect images, videos and sounds to characterize environments and biological interactions. The subfield of animal biometrics develops quantitative approaches to describe and identify species and individuals, using morphological traits and behaviours from audio-visual sources [1]. Examples include species monitoring using audio [2, 3] or visual information [4, 5], identification based on patterns such as color or shape [6], or behaviour from movement trajectories and associated accelerator data [7]. Whereas these approaches show promise for cataloging different aspects of biodiversity [8], they require careful optimization to accurately measure species abundance and phenotypic variation [2].

Digital image and video analysis comprises a set of techniques to perform time intensive tasks, including counting, measuring and tracking individuals [9, 10]. Currently, image analysis is primarily used under controlled laboratory conditions, where populations and individuals can be phenotyped to answer a variety of ecological and evolutionary questions [9, 11]. However, these techniques have also been successfully applied in natural systems, for instance, to identify plankton species in marine surveys [12, 13] and to monitor microorganisms in waste water treatment plants [14, 15]. The wealth of data produced by image and video analysis is both a blessing and a curse. Processing and analysis of the data can become overwhelming when images and videos are constantly taken, and manual steps are needed as to supplement the work flow [1].

Regardless of whether images, videos or sounds are used, making information available requires transforming the raw data (e.g. pixel intensity, movement trajectories or frequency and length of calls) into biologically meaningful information (e.g. number of species observed, the individuals present in a specific area, or behavioural patterns). This transformation can be achieved by machine learning techniques such as classification or regression [16, 17]. Machine learning algorithms use quantitative properties such as the pixel intensity, or features of the objects identified by the image analysis step (e.g. size or shape) to predict the class of an object (e.g. to which species an individual belongs). Supervised learning algorithms are trained on data whose class is known (i.e. labeled) and the goal is to accurately predict unknown (i.e. unlabeled) observations. An important prerequisite for training classifiers is hence that the training data adequately describes the properties of the unknown data.

Populations and communities often show considerable interspecific variation in abundance and intraspecific variation in phenotypic traits [18], both of which may impair reliable species level identification. Phenotypic variation is influenced by intraspecific response to the abiotic and biotic environment, which may induce phenotypic changes in other species within a given community [19]. Predation, for instance, can alter prey size distributions [20, 21], induce the development of defensive traits [22], or induce changes in movement strategies (e.g. emigration, diapause) [23]. Changes in phenotypic expression may also occur as a response to the changes in abiotic environmental conditions or due to species interactions occurring at the same trophic level [22]. Consequently, visual identification methods that rely on phenotypic variation to identify species need to account for dynamic changes in phenotypes to perform accurate classification.

Here we develop, apply and validate a novel framework to automate species classification that accounts for shifting phenotypic traits in response to environmental change. The developed framework focused on accurately classifying individuals to species with diverse phenotypic responses under different abiotic and biotic conditions. We applied and validated our approach using data from a microcosm experiment that assessed changes in species abundances over time in response to temperature and species diversity using a set of six ciliate species. Microcosms are experimental systems, widely used by ecologists and evolutionary biologists, to pose questions about temporal and spatial population and community dynamics [24–26], and have been instrumental in testing ecological theory [24, 27]. Among others, microcosm experiments have been used to investigate the effects of inter- and intraspecific interactions [28, 29] and phenotypic plasticity [30, 31] and hence are a suitable testbed for automated classification of species under variable biotic and abiotic contexts. As our approach considers the dynamic nature of the classification, it complements previous attempts that focused on classification of ciliates in simpler communities without environmental variation [32, 33].

## Materials and methods

### Experimental set up

**Fig 1:**
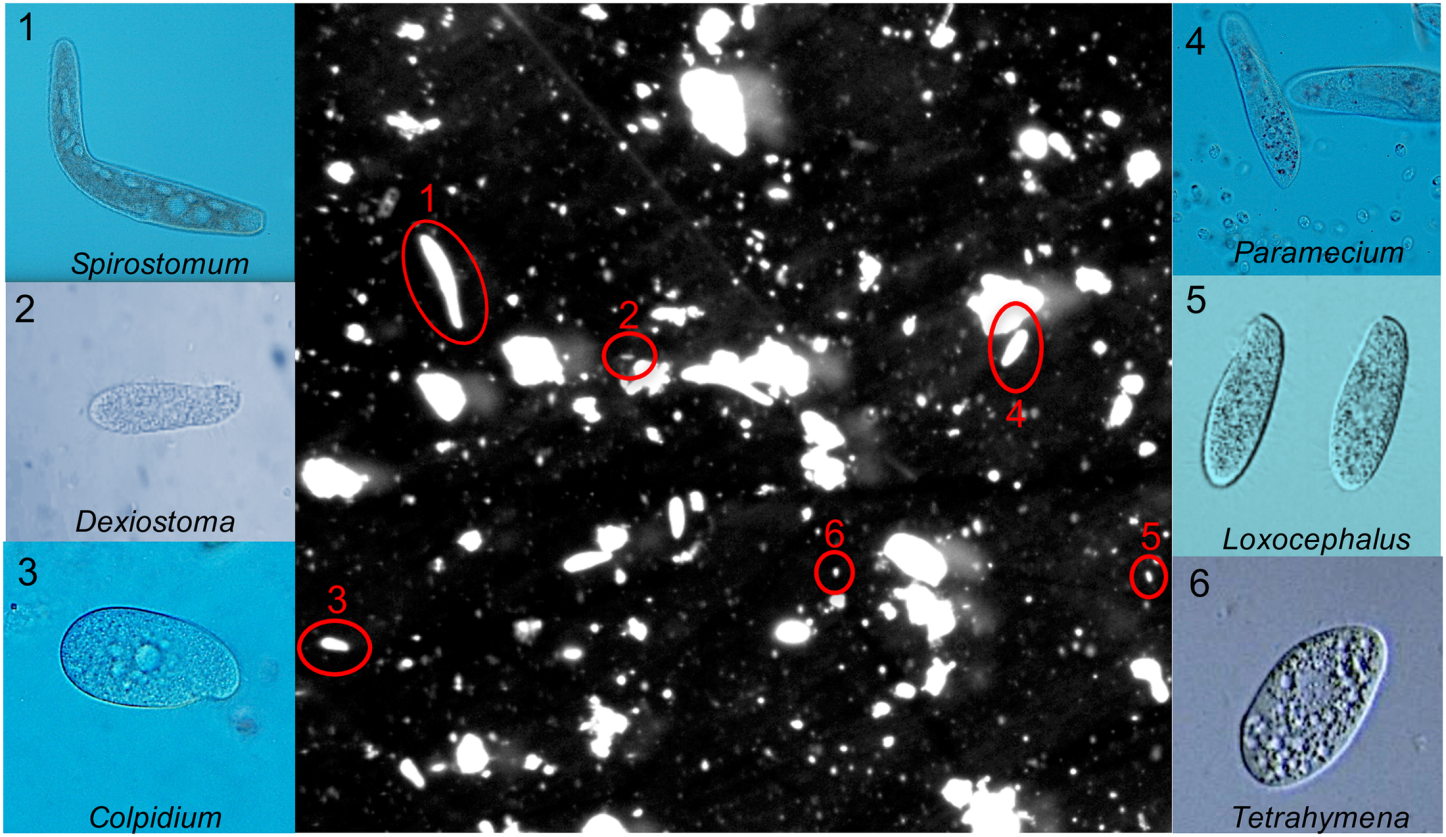
Video frame showing the six ciliate species used in the experiment. The outer images are close-ups of the six species, whereas the center image shows a video frame with all species present at the same scale. Image credits: 1, 3-4: Regula Illi & Florian Altermatt, 2: Michael Fingerle, 5-6: Yuuji Tsukii, available at Protist Information Server, http://protist.i.hosei.ac.jp/.

We used microcosms with six unicellular eukaryotic protist species. All belong to the SAR clade (Stramenopiles, Alveolata, and Rhizaria), further divided into Alveolata and Ciliophora (in the following referred to as ciliates) [34]. The species were *Colpidium striatum*, *Dexiostoma campylum*, *Loxocephalus* sp., *Paramecium caudatum*, *Spirostomum teres*, and *Tetrahymena thermophila* (Fig 1), which are often used in microcosm experiments [24]. The species span a considerable size gradient ranging from ca. 30 μm to 400 μm. Species were selected to be from the same trophic level, hence competing for the same bacterial food resource. None of the species used is able to encyst.

Ciliates were cultured in standard protist medium, along with a common freshwater bacterium, *Serratia fonticola*, as a food source. The organic medium consisted of protist pellets (Carolina Biological Supplies, Burlington, NC) at a concentration of 0.55 g L^−1^ of Chalkley’s medium, and two wheat seeds for slow nutrient release [24].

Two weeks before the start of the experiment, we established fresh ciliate cultures for each species. A sample of 10 mL of stock culture was added to 1000 mL of fresh medium in previously autoclaved 1000 mL glass bottles (GL 45, Schott Duran, Germany). Populations were checked to ensure carrying capacity was reached before the start of the experiment. Single species replicates (species richness 1) were started at a density of three individuals mL^−1^ in 100 mL volume. Multispecies communities were initiated by first making 40 mL of medium from stock cultures. For a species richness of 2, this was made up of 20 mL of each species; for three species there was 13.3 mL of each species, and so on, up to 6.66 mL of each species in six species treatments. This was topped up to 100 mL by addition of 60 mL bacteria inoculated protist medium. The starting densities hence were standardized to a fixed fraction of the species specific carrying capacity, which differed across richness levels (S1 Table). Controls (species richness equaling 0) contained 100 mL of protist medium with *Serratia fonticola*. All cultures communities were kept in 250 mL Duran bottles (microcosms), randomized for each temperature treatment and placed in climate controlled incubators (Pol-Eko Aparatura, Wodzislaw, Poland).

### Experimental design

We used a randomized block design to assess the automated classification of ciliate species across a biotic (i.e. species richness) and abiotic gradient (temperature). Species richness was manipulated across seven levels (0 to 6 species). Since the total number of possible species combinations exceeded the number of feasible units, we randomly selected species combinations for the 3, 4 and 5 species richness levels. The same species richness combinations were repeated in each of the six temperatures. We replicated each level of species richness and composition twice for all levels including an additional replication for the two lowest and the highest levels of complexity (Table 1) resulting in 120 experimental units per temperature (15°C, 17°C, 19°C, 21°C, 23°C and 25°C) and 720 microcosms in total.

**Table 1:** Overview of the experimental design: richness levels, number of unique species combinations per richness level, number of replicates, total of experimental units and inoculum size to start treatments.

richness	unique combinations	replicates	experimental units	inoculum (in ml)
0	1	5	5	0.00
1	6	3	18	< 1.00*
2	15	2	30	20.00
3	10	2	20	13.33
4	15	2	30	10.00
5	6	2	12	8.00
6	1	5	5	6.66

*inoculum size differed among the six species to adjust to density of 3 individuals mL^−1^

### Video sampling, particle tracking and processing

Sampling of each experimental unit occurred every day for the first seven days, then three times per week for the following 50 days and a final sampling seven days later. Sampling took place in two parallel blocks such that half of the experimental units (360 units from three temperatures) were sampled in sequence on consecutive days. For each sampling event, culture medium was gently agitated, and a subsample of 700 μL was taken, mounted onto a glass slide and covered with a glass lid. The height of the sampling chamber was 600 μm and the area filmed 68.7 mm^2^ resulting in 41.2 μL sampled. Five second videos (at 25 frames per second) were taken using 16 × magnification on stereomicroscope (Leica M205 C) mounted with a digital CMOS camera (Hamamatsu Orca C11440, Hamamatsu Photonics, Japan).

We used the BEMOVI package (version 1.0.2) and the statistical computing environment R [35] to process the 18720 videos collected during the experiment and extract the raw trajectories [32]. Global segmentation and tracking parameters were selected for automated processing of videos. The difference lag was defined to be two seconds, particle size was restricted to 20 μm to 8100 μm (corresponding to an input of 5 to 2000 pixel in the BEMOVI locate_and_measure_particle function), and the intensity threshold was set to 10. For particle linking, we specified a link range of 0.12 s (i.e. 3 frames) and a displacement of 81 μm (i.e. 20 pixels). Settings were optimized using a subset of videos (spanning sampling dates of all one, two and six species combinations at 15°C, 21°C and 25°C). Video settings were optimized to err on the side of including false positives rather than exclude true positives at this step, with exclusion of false positives later in the processing pipeline. For further details regarding video processing, please refer to [32].

After tracking, trajectories were filtered to remove noise such as spurious trajectories (e.g. floating debris). Trajectories for analysis were required to show a minimum net displacement of at least 50 μm, a duration greater than 0.2 seconds, a detection rate of 80 percent (for a trajectory with a duration of ten frames the individual has to be detected on at least eight frames), a median step length greater than 2 μm and a minimum mean speed of 50 pixels per second.

### Automated species classification in multi-species communities across temperature environments

**Fig 2:**
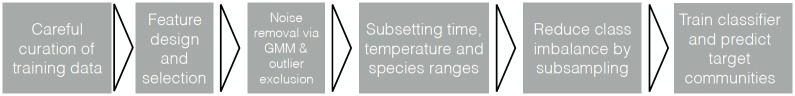
Six steps of the classification pipeline.

In supervised classification, a subset of the data (i.e. training data) with known class assignments (i.e. species identified manually) is used to train the classifier [17]. Training means that the classification algorithm “learns” to distinguish among known classes based on quantitative features such as body size and movement trajectories. The classifier will predict the classes of unknown data (i.e. test data, multi-species communities in our study). Reliable training data is hence crucial to train the classifier and ideally variation in the training data should only stem from biologically meaningful variation. To achieve this, our classification pipeline consists of multiple steps (Fig 2). The first three steps were applied to all videos and can be considered as the data preparation and quality control. The last three steps affect the classification procedure. We tested the sensitivity of our approach using a range of different settings.

#### Careful curation of training data

Microcosms were manually checked for cross-contamination (e.g. ciliates present in controls) during sampling and video recording. If a microcosms was suspected of contamination during the video recording the microcosm was assessed for contamination more stringently, using a stereo microscope. Overall, ten of the 720 of microcosms were contaminated and excluded from further analysis.

A major problem of video analysis is the detection of background particles (e.g. debris) due to movement of the sample liquid (e.g. settling or movement of particles) during videoing. As variation in interspecific ciliate movement is used to classify species, spurious observations due to moving debris will contribute to false positive classifications in the classification pipeline. To mitigate this problem, we first applied more stringent selection for training data. Only trajectories moving faster than 200 μm s^−1^ were included in the training, as debris usually moves more slowly. For classification, all individuals moving faster than 50 μm s^−1^ were considered. Second, we plotted the mean trait values of area and aspect ratio through time to detect outliers and checked these videos manually. After reviewing videos suspected of spurious movements, we excluded inappropriate data. We also defined size boundaries for each species accounting for the change in morphology through time (S2 Table).

#### Feature selection and feature preprocessing

Features are quantitative descriptions used by the classifier to distinguish between the different classes [36]. We identified 10 features informed by our knowledge of distinguishing characteristics across species. The ten features were aggregated at the trajectory level (i.e. mean, standard deviation, minimum or maximum), resulting in 15 features in total (Table 2). These features were a subset of features used in previous classification pipelines [32]. They do not include the advanced movement features (based on wavelet coefficients) as those require substantially longer trajectories for calculation [33].

**Table 2:** Morphological and movement features selected for use in classification.

Code	Type of variable	Measurement method (all are calculated across each of the frames in the particle’s trajectory)
mean_area	Size of particle	Mean area of particle across trajectory
sd_area	Temporal variability in particle size	Standard deviation of particle area
mean_perimeter	Size and shape of particle	Mean length of perimeter of particle
sd_perimeter	Temporal variability of size and shape	Standard deviation of particle perimeter length
mean_major	Length of particle	Mean length of major axes of ellipse fitted to particle
sd_major	Temporal variability in length	Standard deviation of length of major axis
mean_minor	Width of particle	Mean length of minor axis of ellipse fitted to particle
sd_minor	Temporal variability in width	Standard deviation of length of minor axis
mean_ar	Shape of particle	Mean aspect ratio of particle
sd_ar	Temporal variability in shape	Standard deviation of particle aspect ratio
sd_turning	Temporal variability of the direction of movement	Circular standard deviation of particle direction
gross_speed	Particle speed.	Mean of distance travelled between frames
sd_gross_speed	Temporal variability in particle speed.	Standard deviation of distance travelled between frames
max_gross_speed	Maximum particle speed.	Maximum distance travelled between frames
min_gross_speed	Minimum particle speed.	Minimum distance travelled between frames

Features were checked to have non-zero variance and no missing data, scaled to have zero mean and unit standard deviation, and normalized by Box-Cox transformation [37]. Principal component analysis (PCA) was used to reduce the number of features by obtaining uncorrelated principal components [38]. All preprocessing steps were applied to the training data for a given community.

#### Identification and exclusion of noise (using Gaussian mixture models and ellipse fit)

Although the trajectory filtering removed background noise, some spurious trajectories may remain within the training data. To identify spurious trajectories we analyzed the videos from the control microcosms video recordings (0 species richness), which contained no ciliates, hence all detected trajectories are spurious (e.g. due to moving background or changes in light conditions). We trained a Gaussian mixture model (GMM) on the trajectories in the target ciliate culture, and compared these with clusters of spurious trajectories from the control cultures. Clusters that contained mostly spurious trajectories in the ciliate cultures were excluded.

Outliers can have potentially detrimental effects on classification success [37]. Hence, we only included observations in the training data that fell within the 90% confidence ellipse of a bivariate normal distribution, fitted to the first two principal components.

#### Sub-setting species, date and temperature range for training using a sliding window

**Fig 3:**
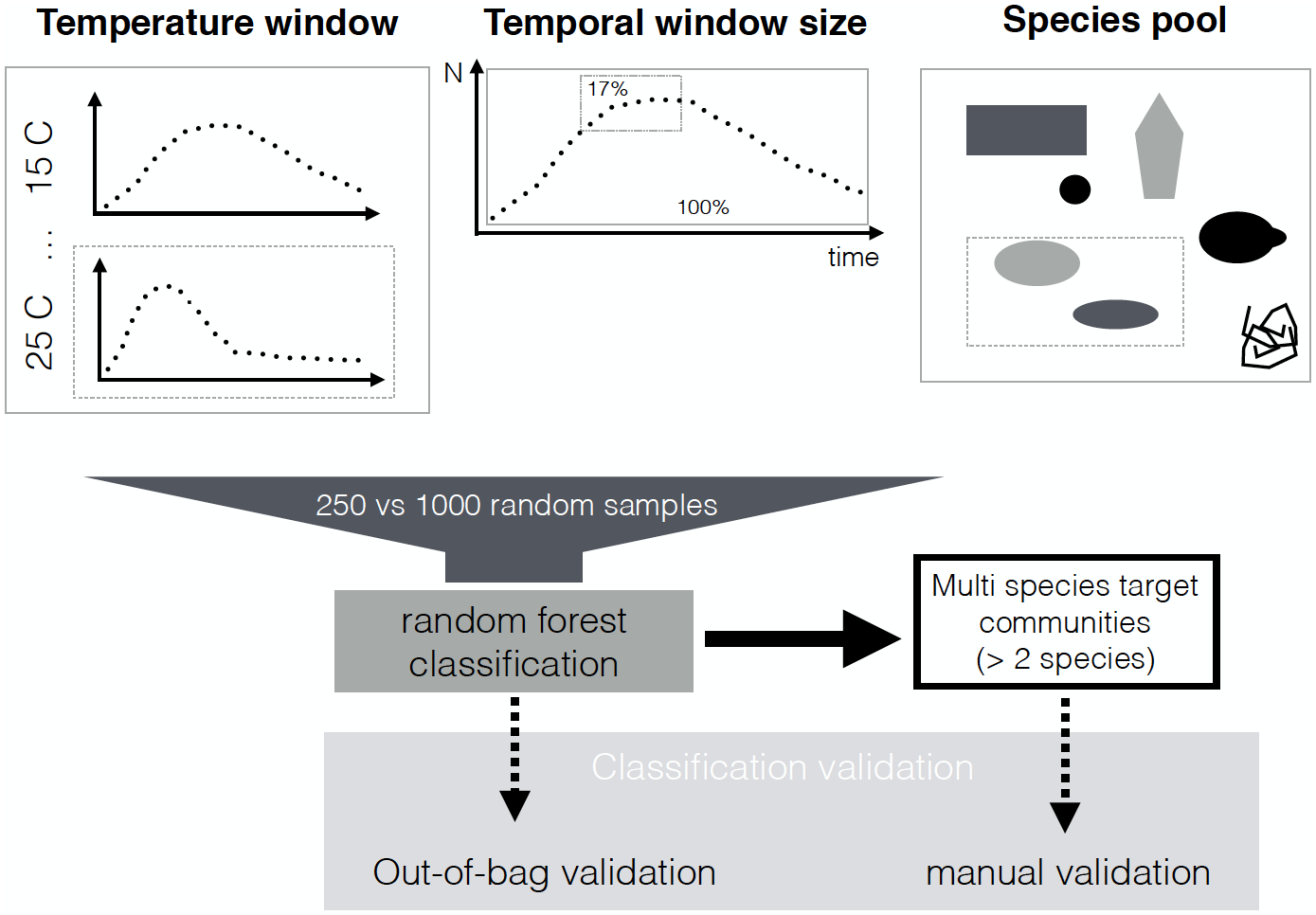
Selecting observations only within certain temperature and temporal distance and using different species pools using the sliding window approach for training the classifier. Imbalance was accounted for via random sub-sampling and the classification results tested using out-of-bag and manual validation.

A major challenge for classifying individuals is that phenotypes change over time and between environments. This creates a dynamic classification context in which individual features of each category vary temporally and across environments. To study the effect of the dynamic context, we first compared models differing in the number of species used for training. We fitted a model, which contained all the species and an additional noise class that represents the spurious trajectories from the controls, yielding a maximum of seven different classes. We also built a customized model only containing the species of the known community and the noise (i.e. information based on the experimental design). Second, we only selected training data within a certain distance in time and temperature of the community to be classified, using a sliding window. We compared different window sizes (10, 30 and 60 days, i.e. 17%, 50% and 100% of the sampling time) and the temperature range (train based on the temperature of target community vs. all available temperatures). Fig 3 summarizes and illustrates the sliding window approach.

#### Reduction of imbalance by randomly selecting observations for training

The ciliates used in the experiment show variation in densities and growth dynamics [24], hence the number of individuals differed among species (i.e. species abundance). Imbalance in the number of observations for different classes in the training data can severely decrease the performance of the classifier for the rare class (e.g. low abundance species) [36]. Various techniques were developed to deal with this important problem [39]. We used a random under-sampling scheme to the majority class to achieve balanced numbers of observations for all classes in the training data. We compared classification success when 250 or 1000 randomly selected observations per species were used.

#### Fitting the random forest classifier

We used the random forest classifier (RF), as it is computationally efficient, and often recommended for yielding reliable results “off-the-shelf” [40]. Naive Bayes and Support Vector Machines were also tested and provided very similar classification success. Random forest is a widely used classification algorithm based on ensembles of decision trees [41]. We used the randomForest package in R for classification [42]. Decision trees are based on binary thresholds that divide observations into classes, with the goal of the purest possible classes at the end nodes. Features of observations whose class is known *a priori* are used to train the classifier. RF is quite robust against over-fitting because only a constrained number of observations and variables is used when building individual decision trees, effectively de-correlating trees within the larger ensemble. Each tree in the ensemble will predict the class of the unknown event and the final class is based on the majority vote of the ensemble [43].

As there was no association between the replicate of the training data and the test data, we pooled replicates of a given community for training and testing. We used an ensemble of 500 decision trees for each ciliate community and one species class was assigned to each trajectory according to the majority vote of the ensemble. At each split the RF classifier choose the square root of available features, and we set the minimum number of observations in each terminal node to one.

### Evaluating automatic classification and sliding window approach using out-of-sample prediction

Out-of-bag classification success in the training data was the response variable used to understand effects of temperature and biotic context. The out-of-bag success states how well the classification model performs on observations not included in training the model (i.e. a out-of-sample prediction) and hence represents an unbiased measure of classification success [41]. Random forest implements a three-fold split validation because only about 66.6% are used for training, while the remaining 33.3% are used for testing. This approach renders additional cross-validation unnecessary for the random forest [41]. We used the proportion of individuals correctly predicted as a given species in the total population as the response.

Generalized linear mixed models were fitted with the lme4 package [44] in R [35]. Predictor variables were centered and scaled to compare effect sizes and to assist numerical convergence of the models. First a model was fit to understand the effect of temperature and species richness on classification success across the full dataset: temperature and richness were modeled as fixed effects; species nested in community composition was included as a random effect to account for the repeated measurements. We also added an individual level random effect (based on the microcosm ID) to account for over-dispersion in the data [45].

To understand the effect of sliding windows, we used contrasts among species pool, temporal and temperature window, as well as the number of randomly sampled trajectories. For these contrasts, only classification success from the pairwise interactions was used as classifying all combinations would haven taken an excessive amount of time (up to a week for each contrast) and hence only using pairwise interactions allowed us to screen the parameter space in a reasonable amount of time. For each of our four sliding windows, we fitted a separate model: temperature and sliding window selection were modeled as fixed effects. As before, species nested in community composition was included as a random effect and an individual level random effect (based on the microcosm ID) accounted for excessive over-dispersion in the data [45].

### Validation against manual classification

In addition, the automatic classification approach was validated against manual species identification by experts. We randomly selected 3 trajectories for each species from richness level 3 using samples from days 14, 25 and 37 after the start of the experiment and from three temperatures (15°C, 21°C and 25°C). Three experts independently assigned individuals (i.e. trajectories) to species using the same videos that were used for automated classification. We used a majority vote (e.g. majority of votes of the different human observers and the automatic identification) as the reference against which we tested the identifications of each expert.

The majority vote was not always unanimous. In 614 of 661 trajectories a majority vote was established, whereas in the remainder no majority vote was found and hence trajectories discarded from further analysis. 43% of these cases were IDs divided between *Tetrahymena/Dexiostoma*, *Dexiostoma/Loxocephalus* (13%) and *Colpidium/Loxocephalus* (11%).

We evaluated the sensitivity and specificity for each species by comparing each expert against the consensus vote using the confusion matrix [46].

In a two species classification, sensitivity is defined as:

$$\frac{\text{number}\;\text{of}\;\text{individuals}\;\text{predicted}\;\text{to}\;\text{be}\;\text{species}\;x}{\text{number}\;\text{of}\;\text{individuals}\;\text{known}\;\text{to}\;\text{be}\;\text{species}\;x}$$ (1)

whereas specificity is defined as:

$$\frac{\text{number}\;\text{of}\;\text{individuals}\;\text{not}\;\text{predicted}\;\text{to}\;\text{be}\;\text{species}\;x}{\text{number}\;\text{of}\;\text{individuals}\;\text{known}\;\text{not}\;\text{to}\;\text{be}\;\text{species}\;x}$$ (2)

## Results

### Video processing and analysis

18 720 videos were taken of which 165 had to be excluded to due to contaminations (resulting in 18 555). 18 320 were successfully processed to provide particle morphology and movement features (example frame S1 Fig). For 235 videos the particle tracking algorithm failed due to excessive amounts of moving debris (many thousands of particles per frame). High particle numbers resulted from directional flow of liquid in the microscope slide caused by improper handling or external disturbances during the video recording. The processed videos provided 1 702 138 177 observations across 43 267 551 trajectories.

### Training data curation

Species richness 1 was used to train the classifier and hence needed to be of the highest quality. We assured this by manual checks on the data and removed an additional 45 videos from the controls, and 164 from species richness 1, resulting in a total of 18 111 videos for the final analyses.

The stronger filtering applied to the training data resulted in removal of 3 897 680 out of 4 214 617 trajectories (92% reduction). Most of the removed trajectories were very short and represented noise. The morphological boundaries for each species (S1 Table) applied to the remaining 316 937 trajectories removed another 22 230 trajectories from species richness 1, leaving 294 707 trajectories to train the classifier.

### Feature selection and preprocessing

**Fig 4:**
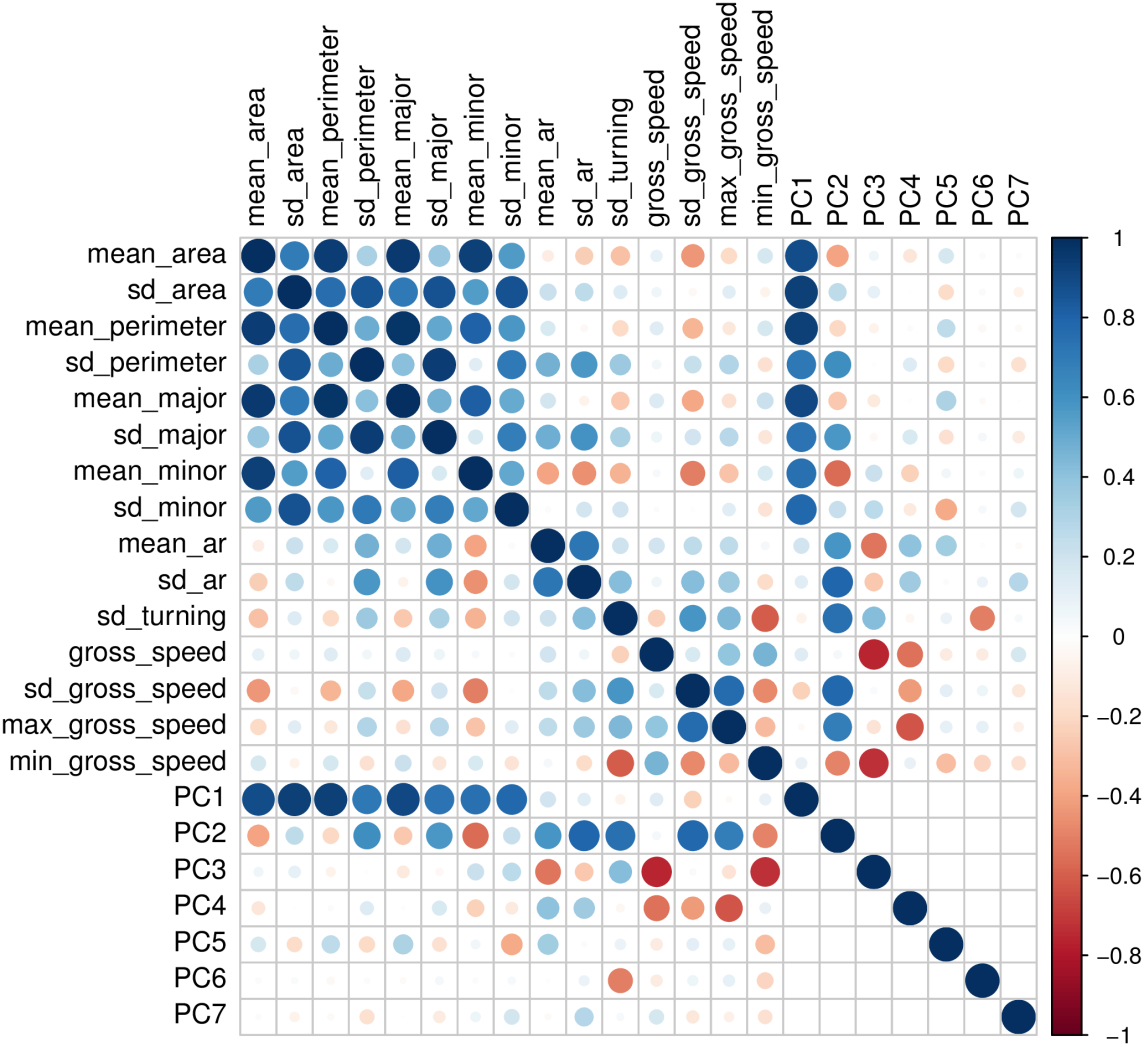
Correlations among original features and principle component scores.

Seven principal components (PCs) accounted for ca. 95% of the variability in the data. PC1 was strongly associated with the eight features relating to cell size, all having positive associations (Fig 4). PC2 was related to variability in turning angles, size and shape. PC3 was strongly negatively associated with speed features. PC4 captured mostly the mean aspect ratio, whereas PC5 to PC7 were only weakly associated with original features.

### Identification and exclusion of noise (using Gaussian mixture models and ellipse fit)

Noise was removed from each subset of data used for training. Trajectories from control cultures (no ciliates present), occupied a distinct area of the PCA feature space (S2 Fig). The presence of spurious trajectories in the ciliate communities created two relatively distinct clusters (e.g. *Paramecium*), which sometimes overlapped with the ciliate cluster (e.g. *Dexiostoma*). Although noise sometimes overlapped with certain species for PC1 and PC2, for other dimensions it may be not overlapping (not shown).

The GMM was able to identify noise and ciliate clusters. Trajectories from the training data (*Tetra* or *Loxo*) falling into areas with spurious trajectories, were re-classified as noise. Reclassification resulted in fewer trajectories from the training data residing in the “noise region” of feature space (S3 Fig). The 90% confidence ellipse fitted around the observations removed additional outliers and improved the species boundaries in multivariate trait space (S4 Fig).

### Effects of temperature, species richness, and sliding window on classification success

**Fig 5:**
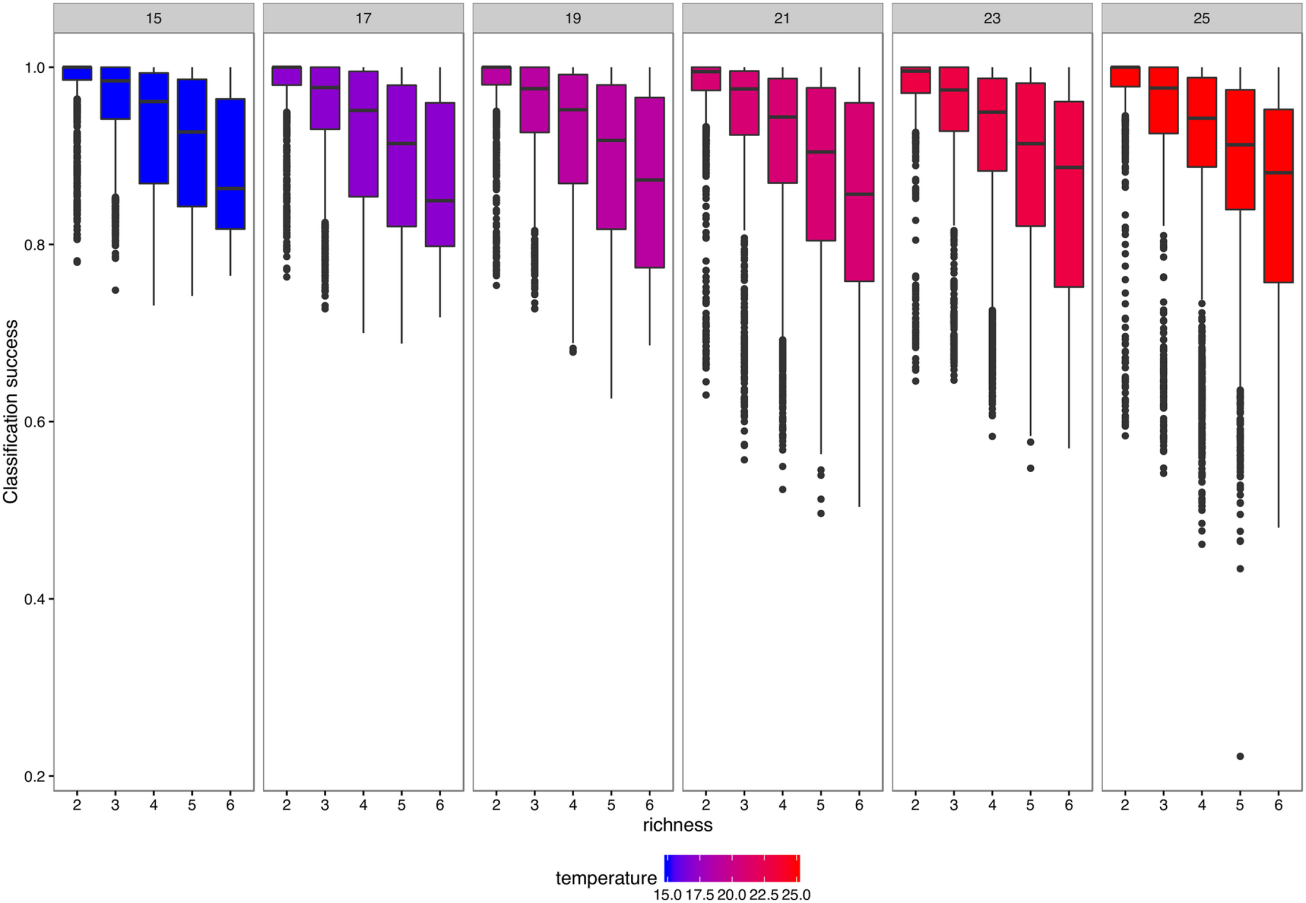
Observed classification success across all temperatures and species richness levels. Species richness (x axis) and temperature (panels) decreased classification success. At higher temperatures, certain combinations drop in classification success resulting in lower classification success.

Overall, increased temperature (b = -0.129, SE = 0.016, p < 0.001) and more species (b = -0.852, SE = 0.119, p < 0.001) decreased classification success across species combinations, whereas their interaction was non-significant (b = -0.012, SE = 0.016, p = 0.47; Table 3). The richness effect was about seven-fold stronger than the temperature effect (Table 3 and Fig 5).

**Table 3:** Model table showing effects of temperature and species richness on classification success.

	Model 0
(Intercept)	3.056(0.119)***
temperature	−0.129(0.016)***
richness	−0.852(0.119)***
temperature:richness	−0.012(0.016)
Num. obs.	24248
Num. groups: ID	282
Num. groups: combination:predicted.species	156
Var: ID (Intercept)	0.063
Var: combination:predicted.species (Intercept)	2.153

***p < 0.001

The contrasts based on sliding windows showed that classification success decreased when all species were included in the training species pool compared to only the known species comprising the community (b = -0.871, SE = 0.003, p < 0.001). Temperature decreased classification further (b = -0.127, SE = 0.023, p < 0.001) and the interaction between temperature and species pool was also negative (b = -0.032, SE = 0.004, p < 0.001; S3 Table).

Increasing the temporal window size decreased classification (b = -0.080, SE = 0.004, p < 0.001), supporting that smaller temporal windows are beneficial because they capture the temporal dynamics. However, the effect was weaker than the temperature effect (b = -0.104, SE = = 0.041, p < 0.05) and not further mediated by temperature (b = -0.0003, SE = 0.004, p = 0.93; S4 Table).

Including more temperatures in the training data decreased classification success (b = -0.072, SE = 0.004, p < 0.001), and the effect size was similar to the temperature effect itself (b = -0.076, SE = 0.019, p < 0.001). The interaction between temperature and number of included temperatures was positive suggesting that these effects cancel out (b = 0.075, SE = 0.005, p < 0.001; S5 Table).

Finally, classification success increased with the number of trajectories included (b = 0.141, SE = 0.004, p < 0.001), whereas the temperature effect was negative (b = -0.135, SE = 0.052, p < 0.001). No interaction effect was found meaning that higher numbers of trajectories generally were beneficial across temperatures (b = 0.006, SE = 0.004, p = 0.11; S6 Table).

### Validation against manual classification

**Fig 6:**
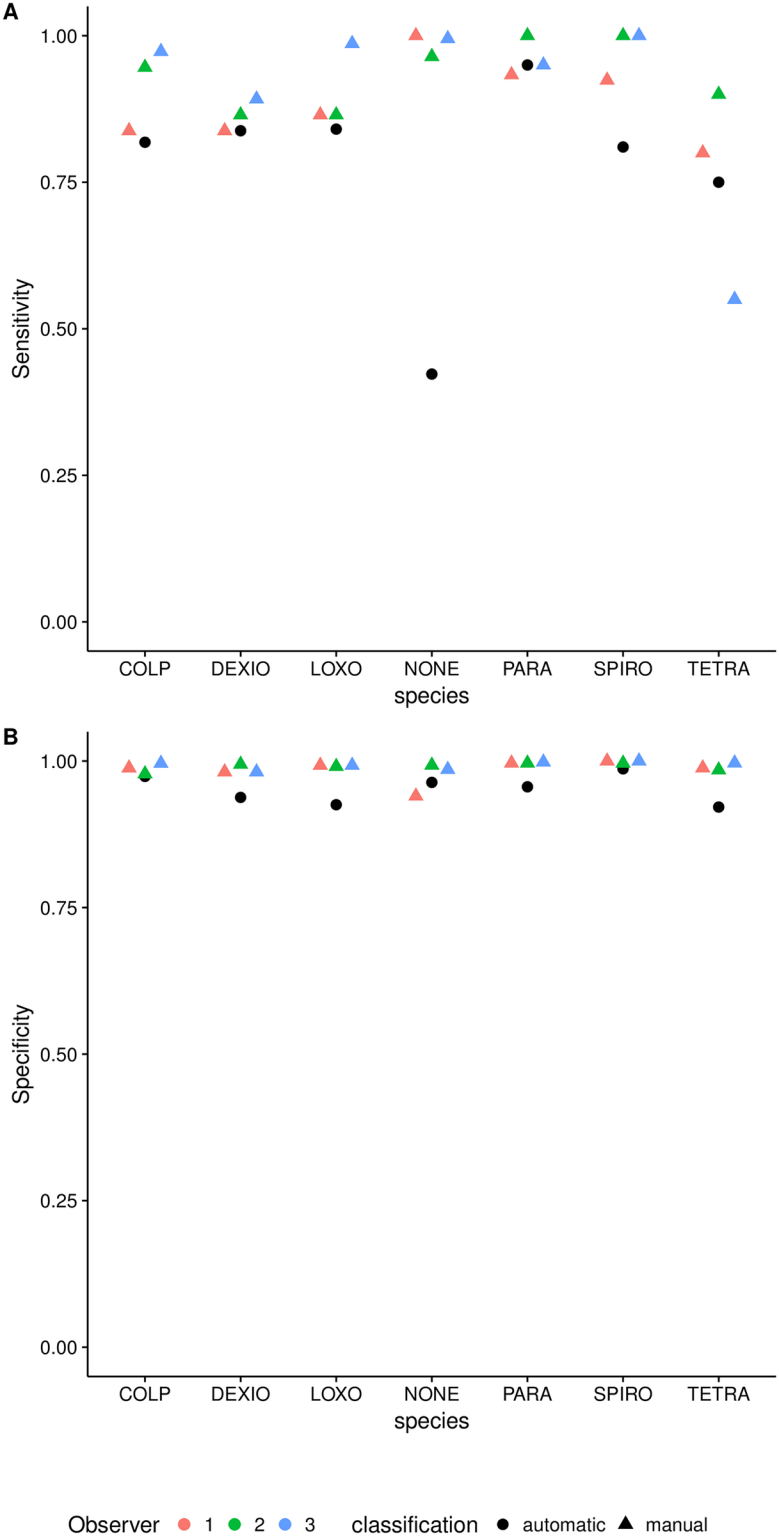
Comparison of manual and automatic classification success for each of the species. Panel A shows the sensitivity, whereas Panel B shows the specificity against the consensus vote. Different colours show different experts, whereas different shapes show manual versus automatic identifications. The automatic classification behaves very similar to the experts both in terms of sensitivity and specificity for the six ciliate species.

When we compared the classification success for automatic and manual classification, we observed high classification success (sensitivity and specificity) for both manual and automatic classification of the six ciliate species (Fig 6). Manual classification is often slightly better than automatic classification, but automatic classification can outperform manual observers for some classes (e.g. *Tetrahymena*) (Fig 6). Although we included trajectories from different combinations and temperatures, species classification success remained above or close to 80%, even for species like *Tetrahymena* whose accuracy was lower in the out-of-bag validation. Furthermore, we found that sensitivity is correlated between manual and automatic classification, i.e. they experience the difficulties with the same species.

Automatic classification did less well in identifying spurious trajectories (i.e. noise), with *Tetrahymena* (21%) and *Dexio* (13%) being the most confounded classes. Although this shows that some noise escapes our cleaning procedure and that the error is non-randomly distributed across species, overall we found a strong positive correlation between automatic and manual counts of ciliates (Pearson correlation coefficient = 0.76).

Support vector machines and Naive Bayes classifier provided very similar classification success and hence are only shown in the appendix (S5 Fig).

## Discussion

Here we introduce a methodological framework to automate species identification from individual phenotypes in dynamic contexts. We show that we can reliably classify species from video recordings using phenotypic variation in morphology and movement, while accounting for environment dependent phenotypic variation. We demonstrate our approach by identifying ciliates in aquatic microcosms, but the techniques generalize to a wide range of study systems, including aquatic algae and zooplankton. Our results highlight the importance of the dynamic context of biotic interactions and abiotic environment for accurate species level classification. Overall, increased temperature and species richness reduced classification success, with species richness inducing an order of magnitude greater decrease than temperature. Importantly, sub-setting training data according to time, temperature and species richness yielded increased classification success.

### The need to account for dynamic trait change in communities

Intraspecific variation in phenotypes is often observed for animals and plants and modified in response to abiotic and biotic environmental factors [18]. The mechanism organisms use to cope with environmental variation is called phenotypic plasticity and allows for changes in morphology, physiology or behavior [47]. We used ciliates as study organisms, as they show large variation in phenotypes in response to environmental factors. For instance, ciliates show smaller body size when exposed increased temperature [48] or change their feeding behavior when predators or competitors are present [49]. Despite such changing phenotypes over time and space, we show that our approach more accurately identifies species. This highlights the importance of accounting for phenotypic variation when identifying species based on morphology [50].

Our approach in turn provides highly resolved time series of population and community dynamics. These can be used to leverage the power of recently developed time series analysis methods for complex, nonlinear systems [51]. A promising avenue for future research is to reconstruct networks of species interactions from such multivariate time series [52, 53].

### Improved trait resolution due to dynamic sliding windows

The sliding window approach outperforms models that include global information (e.g. all species, all sampling dates, or all temperatures) for classification of multi-species communities. For instance, classification success improved when using a sliding window with data from a few sampling dates compared to using data from all sampling dates. Improved classification is a result of selecting training data that focuses on strong environment-dependent changes (due to species interactions and temperature) in traits (i.e. phenotypes). In contrast, when all data were pooled without considering the environment, systematic changes would be swamped by the overall trait variability.

Our finding can be easily illustrated with a trait like body size. Imagine two species that initially differ in mean body size, that shrink through time, but at different rates (i.e. resulting in overlapping body sizes). If all data were pooled, the classifier would consider individuals within the whole range of body sizes observed over the experiment for the two species, resulting in overlapping body size distributions. However, a classifier that only considers body size at a given time point may capture temporally non-overlapping sizes and hence have higher classification success.

### Community context and potential to tell apart similar species

Successful classification was greater when species presences was assumed based on the expected community diversity (e.g. initial microcosm species diversity) in the statistical model compared to assuming all species were potentially present in each community. This is expected, as classifiers tend to perform less well with increasing numbers of classes to predict [37]. The consequence being that if the presence of certain species can be ruled out (as in our experimental design), it is better to work with a more specific classifier. This may not work under non-experimental settings where the expected species are unknown and also has costs in terms of re-training the classifier multiple times. If the species to be expected in a community are unknown, training the classifier on all possible species may be preferable.

Our results also highlighted difficulties in telling the two smallest species apart (i.e. *T. thermophila* and *D. campylum*). This effect was stronger at higher temperatures, likely due to smaller size which is common for many protists species [48]. Taking videos at higher magnification has potential to improve classification as traits (e.g. cell shape) may be better resolved. However, higher magnifications entails a smaller sample volume because the viewing field covers less of the slide. This trade-off therefore needs to be carefully considered.

Another approach to distinguish phenotypically similar species may be active learning [36]. Active learning can improve distinguishing classes by requesting user input on decisive observations. Instead of increasing the total number of observations, the technique identifies critical observations for the definition of classes. Active learning autonomously selects these observations and presents them to the human expert for annotating [54]. The manual validation results from this study show that experts can provide reliable identifications from videos, and the costs of manual identifications may pay off substantially in downstream automated classification success when decisive training observations are manually validated.

### Data curation, cleaning, feature selection and dimension reduction

Much of the data cleaning involved careful validation of the raw data, identifying potential problems with the data and designing steps to subsequently clean the data in a more automated fashion. Fundamentally, the classifier is only as good as the training data, meaning that foremost video quality then quantity is important. Observations accidentally labeled as another class (e.g. spurious trajectories due to moving background) may seriously hamper the classification. Our cleaning pipeline therefore deliberately discarded a large amount of trajectories (>92%). This amount is nevertheless comparable to other automatic classification pipelines, for instance, marine plankton classification, where more than 95% of particles were discarded [55] before target objects are classified.

Reducing the number of features (i.e. explanatory variables) had the advantage of requiring less computing time when training the classifier, especially when predictors are highly correlated and hence contain the same information. An excessive number of features may have also decreased the accuracy of the classification, a phenomenon known as the curse of dimensionality [36]. Albeit RF classification does not require feature reduction and transformations *per se*, better numerical stability is expected when features are on the same scale [37].

### Parameterization of classifier and GMM

The RF classifier often produces reliable results with few tuning parameters [40]. We constructed an ensemble of 500 decision trees which was computationally efficient, although a lower number of trees probably would have been sufficient [56]. We restricted the number of features to choose from as candidates at each split to be two, which is sensible given the rather low number of predictors in our case. Increasing that number may lead to stronger correlation within the ensemble of trees, which in turn could lead to overfitting. Regarding the GMM we could have reduced the number of fitted clusters to improve performance. The GMM was clearly one of the bottlenecks of the analysis pipeline, partly because 40 clusters had to be fitted for each community. However, it was a worthwhile effort as noise was efficiently eliminated from the training data.

### Down-sampling data for training

Randomly sampling a number of trajectories from the subsets (training data) reduces the chance of over-fitting. It also removes bias from the random forest classifier because all classes have similar numbers of observations. This proved to be a problem in previous applications of RF [32, 33], where minority classes often had lower classification success than majority classes. Whereas some classifiers are more robust to imbalance (e.g. support vector machines), here we show that sub-sampling the population circumvents the imbalance issue. However, sufficient observations of the minority class are still needed. Increasing the number of observations increased classification success, though with computational cost. Training the GMM on 1000 instead of 250 trajectories led to a four-fold longer training time in the two species combinations, and potentially much longer training in more species rich communities. In case the observations of the minority class are limiting, it may also require increasing the temporal window with associated decreases in classification. Balancing these factors hence needs careful consideration of the properties of the classification problem studied.

### Speed, size of experiment, scalability, workflow

Video analysis and automated species identification allowed a much larger experiment than could have been achieved with manual methods. Manual identification took 3 to 4 person hours for the limited number of trajectories (<700) in the validation dataset, and hence manual classification of all the trajectories in the dataset would have been impossible (>40 000 000 trajectories). Manual counting of the samples under the microscope can take 5-10 minutes depending on abundance and species richness, requiring up to 120 hours of continuous work on each sampling date, which would not have been feasible. The high person-hour demand associated with extensive multispecies microcosm experiments previously limited sampling to either time series data for one ciliate species across a small number of microcosms (50 to 200) [29, 57–59], or more experimental units (>300) but fewer samplings [60, 61]. Our approach circumvents these logistic limitations. It also collects extensive information on phenotypes that opens microcosm experiments, and other similar study systems, to high-throughput analyses of traits.

### Caveats and limitations

Out-of-bag error rates showed high classification success for ciliate and noise. The manual classification showed that automatic classification is indeed comparable to manual classification for ciliates. However, a certain amount of trajectories identified as noise by the manual observers were classified as ciliates by the RF. A possible explanation is that training data was deliberately processed to contain the most reliable observations of a given class. The strong filtering removed most of the noise, but also ciliate trajectories, probably improving the separation of classes in multivariate trait space. In the test data, no strong filtering was applied to make sure all ciliate trajectories remained, however, this also led to spurious trajectories being identified as ciliates. Whereas some noise was mis-labeled as ciliates, bias in the species counts should overall be negligible for several reasons. First, the strong correlation between observed and predicted counts based on the validation dataset indicates that the automatic classification provides reliable counts. Second, the counts of species in a given community (i.e. species abundances) are based on a weighted average across the video. Most of the trajectories that were mis-classified by the RF were substantially shorter than the correct identifications. Hence the short mis-labeled trajectories will only contribute a small fraction to the overall abundances. Third, the quantification of different error sources allows us to incorporate specific measures of observational error into models to analyze the data in later steps. Our manual validation also showed that human observers do not agree on identifications unanimously, however, the human error is almost never stated nor quantified and hence cannot be considered in subsequent analyses.

## Conclusions

Our framework allows reliable classification of individual organisms into species, despite temporal and environmentally induced trait change. We developed the approach based on videos of ciliate, but the methodology and computational pipelines are general and hence applicable to a wide range of organisms, including monitoring of algae communities and microorganisms in biofuel production [62], tracking plankton diversity in natural freshwater or marine environments [63], or in sewage waters [14, 15].

## Supporting information

S1 Fig A single frame of one video, with particles labeled by their trajectory ID. For each trajectory, we obtained morphology and movement properties that were later used for classification into the respective species.
(JPG)

S2 Fig In each panel, a point is a trajectory, with its position on PC1 corresponding to overall size, and PC2 to variability in size, and turning behaviour. Trajectories from microcosms containing ciliates are shown in black, yellow dots are trajectories from the controls (no ciliates). Panel codes: Colp = *Colpidium striatum*, Dexio = *Dexiostoma campylum*, Loxo = *Loxocephalus* sp., Para = *Paramecium caudatum*, Spiro = *Spirostomum teres*, and Tetra = *Tetrahymena thermophila*.
(PDF)

S3 Fig Scatterplot of trajectories in principal component space from videos of three experimental units. Trajectories reclassified as noise by the Gaussian Mixture Model (GMM) are outlined in black. In this example, only some of the trajectories from the *Tetrahymena thermophila* culture were classified as noise (i.e. are outlined in black and have their colour changed from blue to green. (Tetra = *Tetrahymena thermophila*, none = control (no ciliates), and Loxo = *Loxocephalus* sp.).
(PDF)

S4 Fig Scatterplot of trajectories in principal component space from videos of three experimental units (Tetra = *Tetrahymena thermophila*, none = control (no ciliates), and Loxo = *Loxocephalus sp.)*. A 90% confidence interval ellipse is fitted to each of the three experimental units to identify background noise in component space. The observations that fall outside the confidence ellipses are excluded from the training data.
(PDF)

S5 Fig Sensitivity and specificity of alternative classifiers such as support vector machines (SVM) and Naive Bayes (NB), compared to random forest (RF) and manual classifiers. All provide similar classification success for the ciliate species. SVM and NB are even slightly better than RF in terms of classifying noise.
(TIF)

S1 Table Initial densities (individuals mL^−1^) for different richness treatments. (PDF)

S2 Table Morphological boundaries for training data. (PDF)

S3 Table Output model 1. (PDF)

S4 Table Output model 2. (PDF)

S5 Table Output model 3. (PDF)

S6 Table Output model 4. (PDF)
