# Supplementary figures and images for: Dynamic species classification of microorganisms across time, abiotic and biotic environments—A sliding window approach
Source: PLoS One. 2017 May 4;12(5):e0176682. doi: 10.1371/journal.pone.0176682 (PMC5417602; doi:10.1371/journal.pone.0176682)

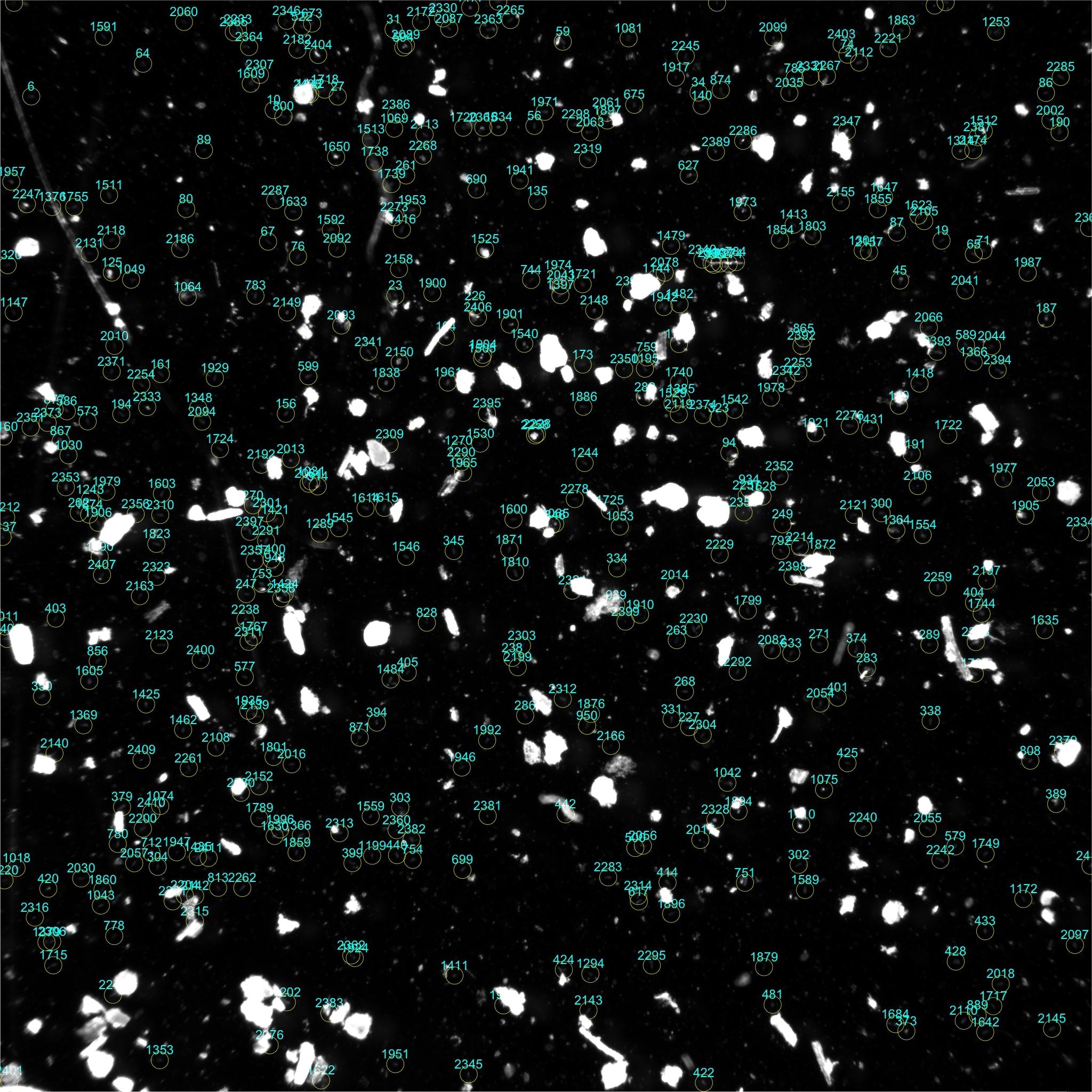

Supplement: S1 Fig — For each trajectory, we obtained morphology and movement properties that were later used for classification into the respective species. (JPG) [file pone.0176682.s001.jpg]

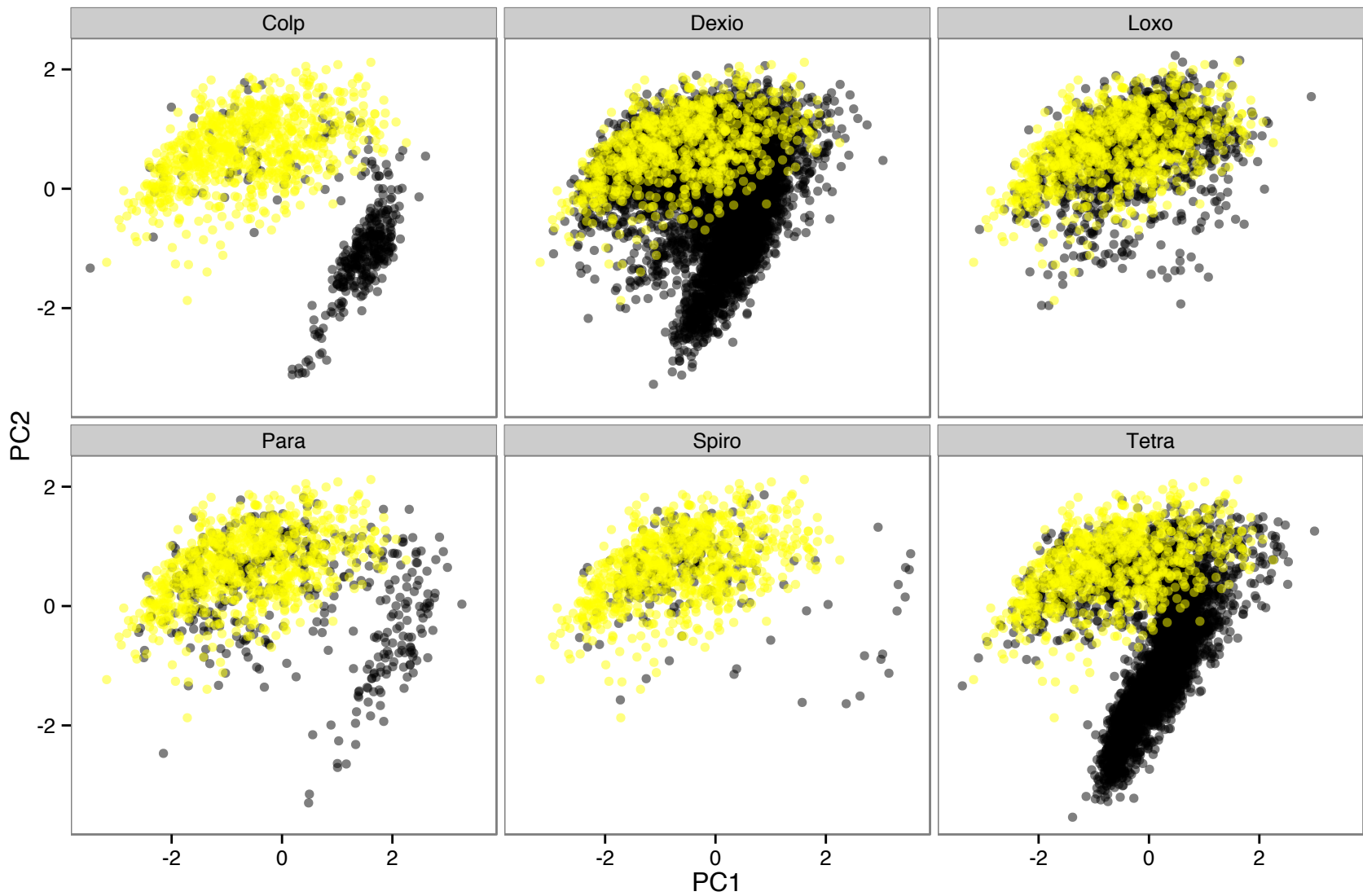

Supplement: S2 Fig — Trajectories from microcosms containing ciliates are shown in black, yellow dots are trajectories from the controls (no ciliates). Panel codes: Colp = Colpidium striatum, Dexio = Dexiostoma campylum, Loxo = Loxocephalus sp., Para = Paramecium caudatum, Spiro = Spirostomum teres, and Tetra = Tetrahymena thermophila. (PDF) [file pone.0176682.s002.pdf]

Data after GMM cleaning

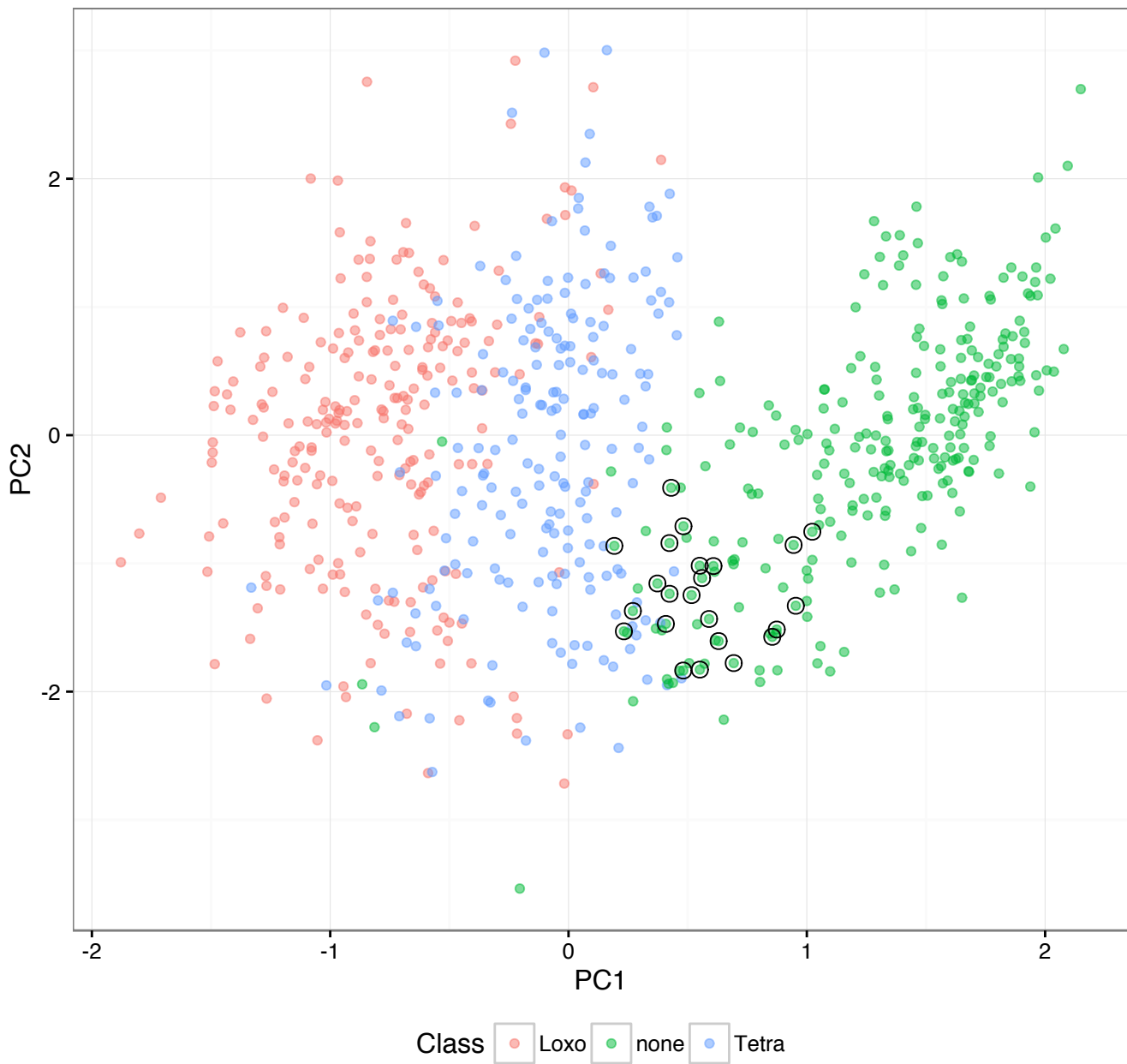

Supplement: S3 Fig — Trajectories reclassified as noise by the Gaussian Mixture Model (GMM) are outlined in black. In this example, only some of the trajectories from the Tetrahymena thermophila culture were classified as noise (i.e. are outlined in black and have their colour changed from blue to green. (Tetra = Tetrahymena thermophila, none = control (no ciliates), and Loxo = Loxocephalus sp.). (PDF) [file pone.0176682.s003.pdf]

Data before outlier removal

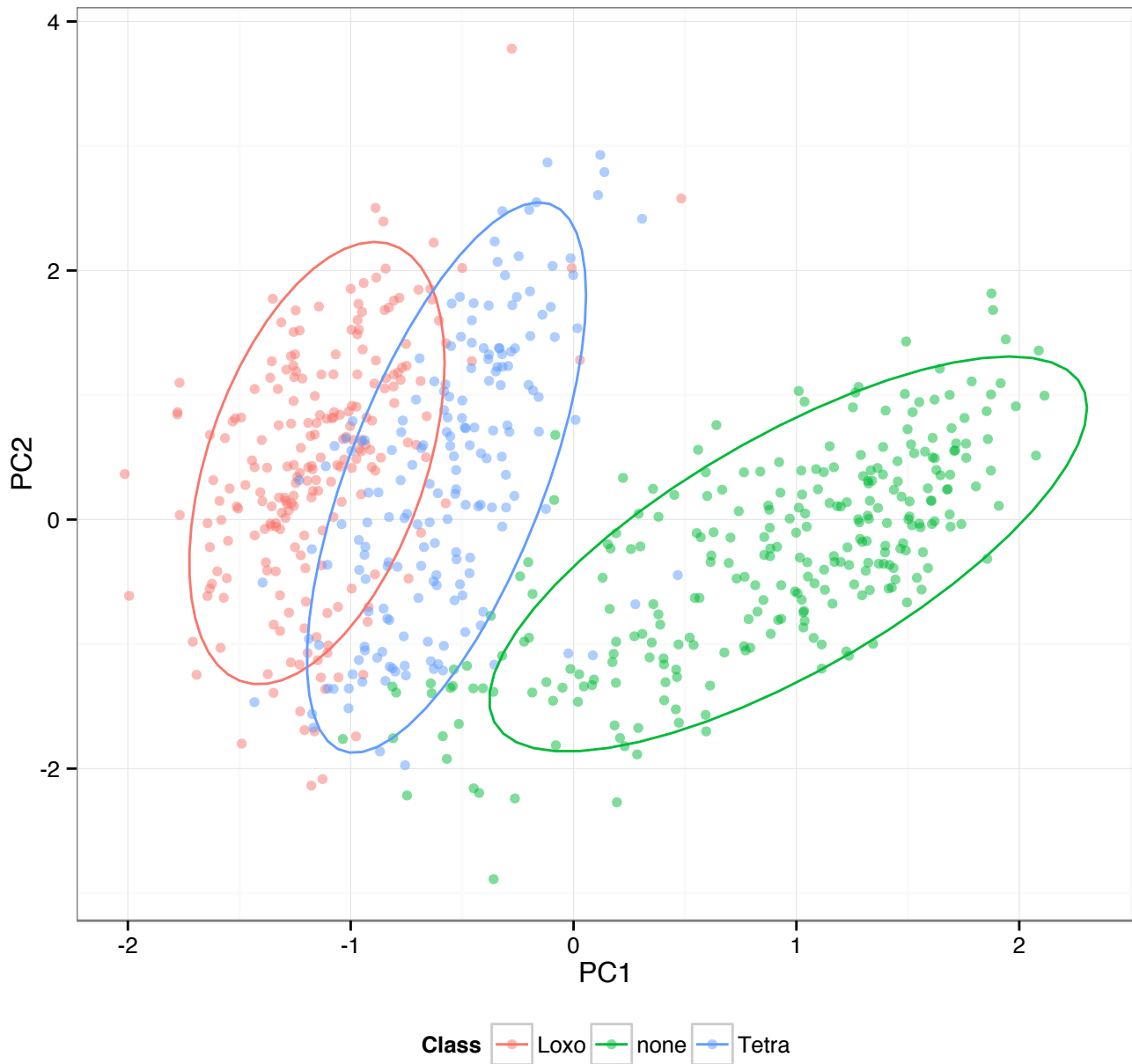

Supplement: S4 Fig — A 90% confidence interval ellipse is fitted to each of the three experimental units to identify background noise in component space. The observations that fall outside the confidence ellipses are excluded from the training data. (PDF) [file pone.0176682.s004.pdf]

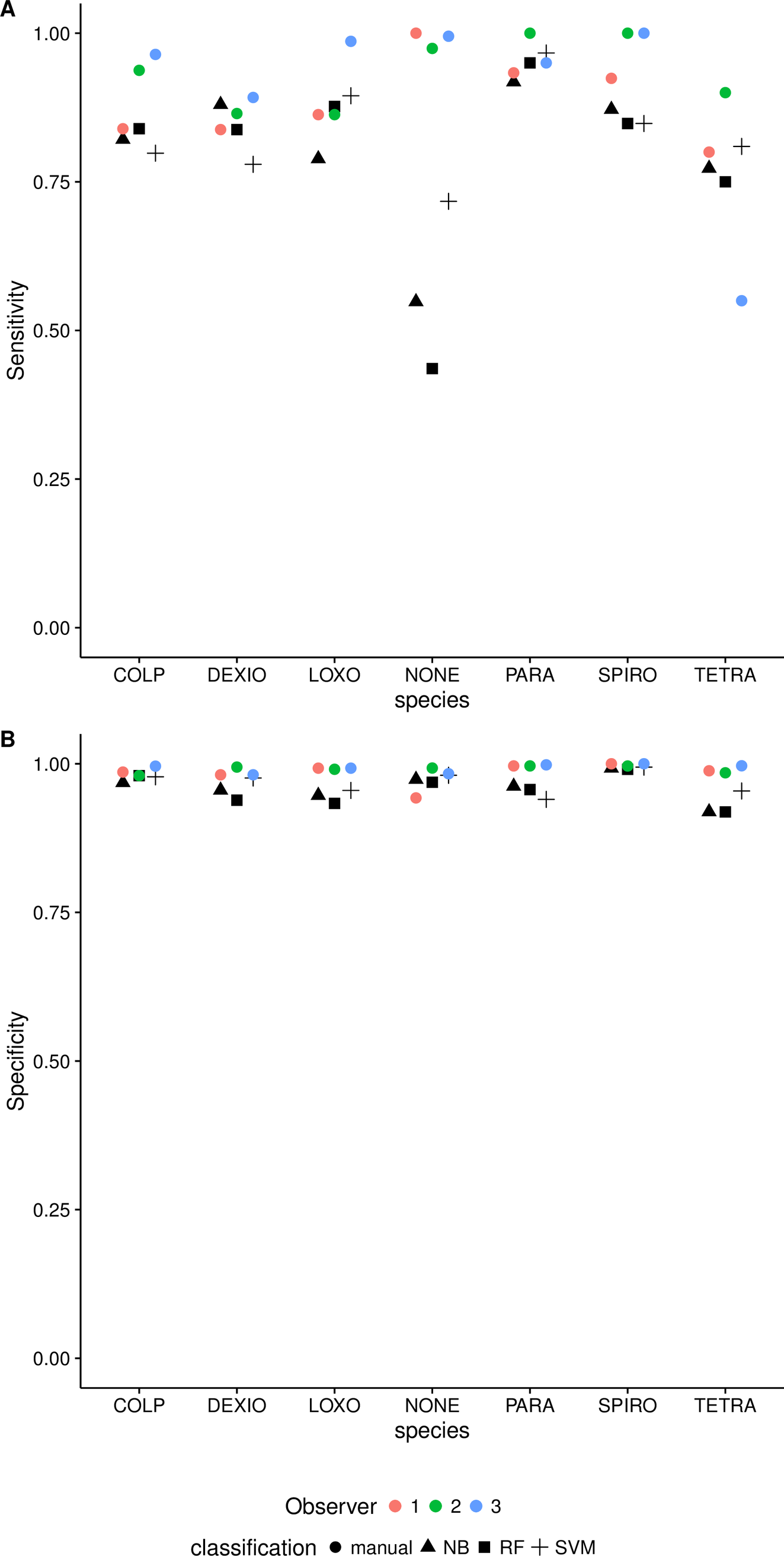

Supplement: S5 Fig — All provide similar classification success for the ciliate species. SVM and NB are even slightly better than RF in terms of classifying noise. (TIF) [file pone.0176682.s005.tif]
