# Supplementary material for: Dynamic species classification of microorganisms across time, abiotic and biotic environments—A sliding window approach
Source: PLoS One. 2017 May 4;12(5):e0176682. doi: 10.1371/journal.pone.0176682 (PMC5417602; doi:10.1371/journal.pone.0176682)
Supplement: S1 Table — (PDF) [file pone.0176682.s006.pdf]

richness	incoculum (mL)	Colp	Dexio	Loxo	Para	Spiro	Tetra
1	-	3	3	3	3	3	3
2	20	249	993	111	121	59	3053
3	13.33	166	662	74	81	39	2035
4	10	124	497	55	61	30	1527
5	8	99	397	44	49	24	1221
6	6.66	83	331	37	40	20	1017
