# Supplementary material for: Dynamic species classification of microorganisms across time, abiotic and biotic environments—A sliding window approach
Source: PLoS One. 2017 May 4;12(5):e0176682. doi: 10.1371/journal.pone.0176682 (PMC5417602; doi:10.1371/journal.pone.0176682)
Supplement: S2 Table — (PDF) [file pone.0176682.s007.pdf]

Species	minimum size	maximum size
Colpidium	100	1500
Dexiostoma	10	500
Loxocephalus	100	1000
Paramecium	100	3000
Spirostomum	1000	6000
Tetrahymena	50	500
