# Supplementary material for: Dynamic species classification of microorganisms across time, abiotic and biotic environments—A sliding window approach
Source: PLoS One. 2017 May 4;12(5):e0176682. doi: 10.1371/journal.pone.0176682 (PMC5417602; doi:10.1371/journal.pone.0176682)
Supplement: S3 Table — (PDF) [file pone.0176682.s008.pdf]

	Model 1
(Intercept)	3.825 (0.261)***
spec_number_included_	−0.871 (0.003)***
temperature	−0.127 (0.023)***
spec_number_included_:temperature	−0.032 (0.004)***
Num. obs.	13998
Num. groups: ID	90
Num. groups: combination:predicted.species	45
Var: ID (Intercept)	0.048
Var: combination:predicted.species (Intercept)	3.031

$^{***}p < 0.001$, $^{**}p < 0.01$, $^{*}p < 0.05$
