# Supplementary material for: Dynamic species classification of microorganisms across time, abiotic and biotic environments—A sliding window approach
Source: PLoS One. 2017 May 4;12(5):e0176682. doi: 10.1371/journal.pone.0176682 (PMC5417602; doi:10.1371/journal.pone.0176682)
Supplement: S4 Table — (PDF) [file pone.0176682.s009.pdf]

	Model 2
(Intercept)	5.810 (0.413)***
window_size_	−0.080 (0.004)***
temperature	−0.104 (0.041)*
window_size_:temperature	−0.000 (0.004)
Num. obs.	20997
Num. groups: ID	90
Num. groups: combination:predicted.species	45
Var: ID (Intercept)	0.139
Var: combination:predicted.species (Intercept)	7.538

$^{***}p < 0.001$, $^{**}p < 0.01$, $^{*}p < 0.05$
