# Supplementary material for: Dynamic species classification of microorganisms across time, abiotic and biotic environments—A sliding window approach
Source: PLoS One. 2017 May 4;12(5):e0176682. doi: 10.1371/journal.pone.0176682 (PMC5417602; doi:10.1371/journal.pone.0176682)
Supplement: S5 Table — (PDF) [file pone.0176682.s010.pdf]

	Model 3
(Intercept)	5.815 (0.434)***
temp_number_included_	−0.072 (0.004)***
temperature	−0.076 (0.019)***
temp_number_included_:temperature	0.075 (0.005)***
Num. obs.	13998
Num. groups: ID	90
Num. groups: combination:predicted.species	45
Var: ID (Intercept)	0.026
Var: combination:predicted.species (Intercept)	8.272

***$p < 0.001$, **$p < 0.01$, *$p < 0.05$
