# Supplementary material for: Dynamic species classification of microorganisms across time, abiotic and biotic environments—A sliding window approach
Source: PLoS One. 2017 May 4;12(5):e0176682. doi: 10.1371/journal.pone.0176682 (PMC5417602; doi:10.1371/journal.pone.0176682)
Supplement: S6 Table — (PDF) [file pone.0176682.s011.pdf]

	Model 4
(Intercept)	6.494 (0.467)***
trj_number_included_	0.141 (0.004)***
temperature	-0.135 (0.052)**
trj_number_included_:temperature	0.006 (0.004)
Num. obs.	13998
Num. groups: ID	90
Num. groups: combination:predicted.species	45
Var: ID (Intercept)	0.227
Var: combination:predicted.species (Intercept)	9.650

$^{***}p < 0.001$, $^{**}p < 0.01$, $^{*}p < 0.05$
